# Supplementary material for: Intolerance of uncertainty and mental health in China “Post-pandemic” age: The mediating role of difficulties in emotion regulation
Source: PLoS One. 2024 Feb 1;19(2):e0298044. doi: 10.1371/journal.pone.0298044 (PMC10833548; doi:10.1371/journal.pone.0298044)
Supplement: Attachment — Submitted filename: Response to Reviewer 1 Comments.docx [file pone.0298044.s001.docx]

**Response to Reviewer 1 Comments**

Dear reviewer 1:

Thank you very much for your time involved in reviewing the manuscript and your comments have further improved the quality of the manuscript.

We have carefully reviewed the comments and revised the manuscript accordingly. The modified section was already highlighted in yellow. Hope the explanation has fully addressed all of your concerns. Point-by-point response to reviewer are attached below this letter.

Please see the attachment.

**Point 1:** The participants are not described in the introduction. I would recommend adding a summary table of the sociodemographic characteristics of the study participants.

**Response 1: Many thanks for your comments, we have added a summary table of the sociodemographic characteristics of the study participants in the Results section (Table 1), for specific modifications see lines 246-252 of the manuscript.**

**Point 2:** The authors should add more details on sample selection. It is not completely clear how was it selected. Additionally, the authors do not propose a minimum sample size, and it is not clear what the reference population is?

**Response 2: Thank you for your suggestions. We have added details about the sample selection and the reasons for the selection, see lines 186-201 of the manuscript for specific corrections.**

**Also, we have supplemented the criteria for minimum sample size, see lines 204-208 of the manuscript for specific modifications.**

**Point 3:** The authors found a very low correlation of(- .09) between IUC and MH. Although it turned out to be significant, the assumed causal relationship that IUC led to MH seems questionable. The authors should emphasize that in the discussion and underline the need of further research to analyze the strength of these relationships.

**Response 3: Your advice makes a lot of sense. We speculate that the low correlation between IUC and MH also constitutes a realistic reason for the complete mediating role of DER between IUC and MH in this study. Therefore, we have supplemented and explained this reason in the Discussion section, as detailed in lines 338-342 of the manuscript.**

**Furthermore, since this study was a cross-sectional design, a causal relationship was not assumed. This is mentioned in the Limitations section (as detailed in lines 382-384). At the same time, we also underlined the need for further research to analyze the strength of these relationships in the Research limitations section, as detailed in lines 388-391 of the manuscript.**
