# Supplementary material for: Intolerance of uncertainty and mental health in China “Post-pandemic” age: The mediating role of difficulties in emotion regulation
Source: PLoS One. 2024 Feb 1;19(2):e0298044. doi: 10.1371/journal.pone.0298044 (PMC10833548; doi:10.1371/journal.pone.0298044)
Supplement: Attachment — Submitted filename: Response to Reviewer 3 Comments.docx [file pone.0298044.s002.docx]

**Response to Reviewer 3 Comments**

Dear reviewer 3:

Thank you very much for your time involved in reviewing the manuscript and your comments have further improved the quality of the manuscript.

We have carefully reviewed the comments and revised the manuscript accordingly. The modified section was already highlighted in yellow. Hope the explanation has fully addressed all of your concerns. Point-by-point response to reviewer are attached below this letter.

Please see the attachment.

**Point:** The data set that is made available is not intelligible. The columns of the table are not in English and the meaning of the values is not explained. Please add a description of the data set where it is made available and use English for the header in order to make it usable.

**Response: Many thanks for your comments. We have unified the abbreviations in the data with those within the manuscript and changed them to English. Meanwhile, we also submitted a "Data availability statement" with this statement as a detailed description of the data set and the meaning of these abbreviations.**

**The data used in this paper and the description of the data set are openly available at the OSF site for this paper at https://osf.io/fme5s/, DOI 10.17605/OSF.IO/FME5S.**
